# Efficacy of Epidural Pulsed Radiofrequency Treatment in Persistent Spinal Pain Syndrome: A Prospective Clinical Study

**DOI:** 10.1155/prm/6200102

**Published:** 2025-04-11

**Authors:** Burak Erken, Gunay Yolcu, Tuba Tanyel Saracoglu

**Affiliations:** ^1^Division of Pain Medicine, Department of Anesthesiology and Reanimation, University of Health Sciences Basaksehir Cam and Sakura City Hospital, Istanbul, Türkiye; ^2^Department of Pain Medicine, Ege University, Izmir, Türkiye; ^3^Department of Pain Medicine, Basaksehir Cam and Sakura City Hospital, Istanbul, Türkiye

**Keywords:** chronic pain, epidural injection, pain assessment, pulsed radiofrequency treatment

## Abstract

**Background:** Persistent spinal pain syndrome type-2 (PSPS-II) is a chronic condition that is characterized by severe pain and results in disability and a significant reduction in quality of life. Despite the wide range of interventional pain treatments that are applied, depending on the complexity of the etiology, epidural pulsed radiofrequency (EPRF) application has emerged as an approach that has gained popularity in recent years.

**Objective:** The objective of this study is to examine the efficacy of EPRF in patients diagnosed with PSPS-II.

**Methodology:** In this prospectively designed study, patients with PSPS-II who had not responded to conservative treatments and epidural steroid injections were subjected to fluoroscopy-guided EPRF. Patients were evaluated with the Numeric Rating Scale (NRS) for pain severity and the Douleur Neuropathique-4 (DN-4) questionnaire for presence of neuropathic pain before the procedure and at one and three months after. Although the change in NRS score was established as the primary outcome measure, the change in the number of patients with neuropathic pain according to the DN-4 was determined as the secondary outcome measure.

**Result:** In the final analysis, data from 42 patients were evaluated. The analysis of the time-dependent change in NRS revealed a statistically significant reduction in the scores for the first and third months, in comparison with the initial measurement. A significant decrease was observed in the number of patients diagnosed with neuropathic pain according to the DN-4 questionnaire in the first month, in comparison with the baseline. However, no significant change was noted in the third month.

**Conclusion:** The utilization of EPRF for the treatment of chronic radicular pain in the setting of PSPS-II appears to be effective in the short term. Further studies are required to ascertain its long-term effects.

**Trial Registration:** ClinicalTrials.gov identifier: NCT06239857

## 1. Introduction

Persistent spinal pain syndrome type-2 (PSPS-II), formerly failed back surgery syndrome, is a chronic condition that is characterized by severe pain and results in disability and a significant reduction in quality of life [1, 2]. The etiologic complexity including the combination of biological, psychological, and social factors contributes to the development of the pain process requiring an interdisciplinary approach to management [3]. Given that low back pain represents one of the most significant causes of chronic pain and the number of spine surgeries has risen in recent times, it is evident that the number of PSPS-II cases is on the rise despite technological advancements [4].

Interventional pain procedures have a significant role in the multimodal treatment plan of patients diagnosed with PSPS-II. A variety of interventional treatment modalities can be employed, including epidural injections, radiofrequency applications targeting specific spinal structures (dorsal root ganglion (DRG), exiting spinal nerves, or other primary sensory afferents in the epidural area), adhesiolysis, and spinal cord stimulation [5]. Radiofrequency treatment, which can be applied in two ways, namely, conventional or pulsed, has been demonstrated to be an effective treatment for chronic low back pain [6]. This occurs through the targeting of various structures, including the facet joint, median nerve branch, DRG, and epidural area [7]. The existence of numerous concomitant etiologies, including adjacent-level disease of either discogenic or facetogenic origin, persistent or recurrent neural compression, and fibrosis, necessitates a patient-specific evaluation of all interventional methods [8].

Epidural pulsed radiofrequency (EPRF) application has emerged as a prominent technique in recent years, with an increasing frequency of utilization [9–12]. Despite the existence of discrepancies in the methodology of application, the epidural space is accessed via the caudal route, and the electrode is positioned in the targeted region under the guidance of fluoroscopy or ultrasonography. A review of the literature reveals that EPRF application is an efficacious treatment for pain and functionality in patients with chronic low back pain, including those diagnosed with PSPS-II [9–11]. In addition to the existing studies, there is a paucity of data pertaining to the multidimensional assessment of pain. The objective of this study was to assess the efficacy of EPRF in alleviating pain, including neuropathic pain, in patients diagnosed with PSPS-II.

## 2. Materials and Methods

### 2.1. Study Design and Population

This prospectively designed study was conducted in a tertiary center hospital between January and October in 2024. The study population comprised patients aged between 18 and 80 years who had undergone at least one surgical procedure for lumbar disc herniation and who had received at least one epidural steroid injection for the treatment of persistent radicular pain, with no beneficial outcome. The primary concerns regarding the inclusion criteria were identified as the presence of radicular pain radiating to the lower extremity, with or without concomitant back pain, and a pain severity rating of at least 4 on the 10-point Numeric Rating Scale (NRS).

The study excluded individuals who had undergone surgery for an etiology other than lumbar disc herniation, those with concomitant lumbar spinal stenosis, spondylolisthesis, fracture, infection, and malignancy findings, those with endocrine, rheumatological, and other systemic diseases that could affect the spine, pregnant women, those with a history of coagulopathy, those with a mental disorder that would affect the evaluation, and those with an allergic history to the drugs to be administered.

This research has been approved by the authors' affiliated institutions (Basaksehir Cam and Sakura City Hospital, date: 18.01.2024, number: 27.12.2023.700). The study was also registered in the clinical trial registration system. Verbal and written informed consent was obtained from all patients participating in the study.

### 2.2. Procedures

**Figure 1:**
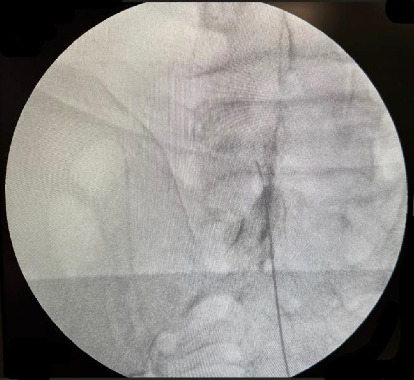
Epidural pulsed radiofrequency treatment. The left L5 level was reached with the RCE catheter, and epidural placement was confirmed with the aid of contrast.

All patients underwent EPRF procedure. The target spinal levels at which EPRF was applied were determined on the basis of the patient's anamnesis, physical examination, and imaging findings. All EPRF procedures were conducted by an experienced pain medicine specialist in a sterile environment, under the guidance of C-arm fluoroscopy. The patients were conveyed to the operating room, where they were positioned prone on the fluoroscopy table and monitored. The injection site was sterilized and a covering applied. The procedures were performed utilizing a 20-gauge, 15-mm active tip radiofrequency needle (Boston Scientific RCE catheter). The epidural space was accessed via a caudal approach, with the sacral hiatus being identified and the targeted level being reached using concomitant fluoroscopy imaging (Figure 1). Following the visualization of the epidural placement of the needle at the targeted level, sensory and motor stimulation was provided to further confirm the level and determine the proximity to the DRG. Subsequently, a pulsed radiofrequency (PRF) application was conducted utilizing a radiofrequency signal generator, with the PRF parameters set as follows: maximum temperature 42°, 45 V, duration 6 min, pulse rate 2 Hz, and pulse width 20 ms. After PRF application, a mixture of 8 mg of dexamethasone disodium, 1 mL of 2% lidocaine, and 1 mL of 0.9% physiological saline solution was administered into the relevant area. The patient was observed for 2 h to check for any side effects.

### 2.3. Outcome Measures

The clinical and demographic data of all patients were recorded, including age, height, weight, duration of symptoms, medications used, and any accompanying diseases. The NRS was employed to evaluate the intensity of pain, while the Douleur Neuropathique-4 (DN-4) questionnaire was utilized to ascertain the presence of neuropathic pain.

NRS: The NRS is a frequently used method for measuring pain severity and monitoring progress. The scale comprises 11 points, ranging from 0 for no pain to 10 for the most severe possible pain. Patients are asked to rate their pain between 0 and 10.

DN-4 questionnaire: The DN-4 questionnaire is a simple and rapid to administer diagnostic tool employed for the identification and assessment of neuropathic pain. Neuropathic pain–related 10 items were grouped under 4 questions. 7 items are derived from the patient's description of their pain, while 3 items are based on the sensory examination conducted by the clinician [13]. A score of 4 or more suggests the presence of neuropathic pain [13].

The data obtained from the NRS and DN-4 assessments were recorded at the preintervention period and at first and third months post-intervention follow-up. All evaluations were conducted by a separate clinician, who was not involved in the procedure. While the change in NRS score was established as the primary outcome measure, the change in number of the patient with neuropathic pain according to the DN-4 was determined as the secondary outcome measure.

### 2.4. Statistical Analyses

SPSS Version 23.0 (IBM Corp., Armonk, NY) program was used for statistical analysis. A power analysis indicated that a minimum of 34 patients should be included in the study, according to the anticipated change in the NRS score at three months determining *α* of 0.05 and a power of 0.80 [9]. The normal distribution of the data was evaluated with the Shapiro–Wilk test. While the one-way repeated measures ANOVA test was used to evaluate whether there was a significant change in the NRS score during the follow-up periods, the Friedman test was used for DN-4. The significance level was set at *p* < 0.05.

## 3. Results

In the final analysis, data from a total of 42 patients were evaluated.  Table 1 presents the demographic data and initial clinical features of the patients.  Table 2 provides the features of lumbar disc herniation surgery and EPRF.

The analysis of time-dependent change in NRS revealed a statistically significant reduction in the scores for the first and third months, compared to the initial measurement (Table 3). A significant decrease was observed in the number of patients diagnosed with neuropathic pain according to the DN-4 scale in the first month, compared to the baseline (Table 3). However, no significant change was noted in the third month (Table 3).

In the course of the EPRF procedure, four patients exhibited transient hypotension, which was successfully managed with basic supportive measures. A uniform sedation procedure was administered to all patients, and transient hypotension was evaluated in relation to patient-related factors such as vulnerability to vasovagal reactions and preprocedural hydration status. Three patients experienced transient increase in pain following the procedure. No significant adverse effects or complications were observed in any of the patients.

## 4. Discussion

Based on the results of this study, it was concluded that EPRF application could be effective option for pain relief in patients with PSPS-II. To our knowledge, this study contributes to the literature by being the first to prospectively evaluate the effect of EPRF application on multidimensional pain assessment in patients diagnosed with PSPS-II.

PRF applications have gained considerable popularity in recent years, becoming an effective treatment option for patients with chronic pain [14]. Although the relatively long duration of action and the low complication rate due to the non-neuroablative nature of the technique can be regarded as advantages, it is important to highlight that the precise mechanism of action remains unclear and that the evidence supporting its efficacy is currently insufficient. A further point that requires elucidation with regard to PRF applications, including our study, is the determination of optimal radiofrequency parameters. In light of the aforementioned circumstances, an increasing number of studies have been conducted in recent years, as evidenced in the literature on high-voltage PRF applications. These studies have yielded findings suggesting that high voltage may be a more effective approach [15]. In addition to the effectiveness of high voltage, further examination is required of other parameters such as PRF duration and resistance. This will facilitate the determination of the optimal application through further studies. It would be beneficial to assess the findings of our study in conjunction with those of other future studies on radiofrequency parameters.

In the context of radicular pain, including PSPS-II, the DRG and the epidural space containing exiting spinal nerves or other primary sensory afferents represent a significant treatment target. While the evidence remains inconclusive, the growing body of literature on the use of PRF techniques targeted these anatomical structures is a promising development [16]. Although there is evidence indicating that PRF application to the sacral epidural area with a caudal approach under fluoroscopy or ultrasound guidance is beneficial in approximately one-third of patients [9, 10], the effectiveness of this approach remains controversial due to the treatment target being mainly in the sacral region. Another, and possibly the most frequently utilized PRF application in clinical practice, is the transforaminal approach to the DRG, which can be performed with or without an epidural steroid or local anesthetic injection [17, 18]. Although the efficacy of this approach has been demonstrated in several studies, one of the most significant disadvantages is the necessity for separate punctures at each target DRG level, which may result in reduced patient comfort.

An alternative approach to DRG is the application of PRF treatment to the target DRG levels from epidural space, with access gained to the epidural area via the caudal route, which is the same method employed in the present study. As previously stated, other neural structures within the epidural space that are targeted by PRF include exiting spinal nerves and sensory afferents. In addition to the direct targeting of neural structures, the immunological effects of PRF in the epidural area may also play a role. Although this approach has numerous theoretical advantages, there is a paucity of data in the existing literature. In a study conducted by Vigneri et al., following the administration of PRF treatment to patients with chronic lumbosacral neuropathic pain utilizing a multifunctional catheter, a minimum 50% reduction in pain scores was observed in 57% of patients at 1 month and 48% at 6 months [19]. The principal distinction between our study and theirs is that patients in the latter group underwent additional epidural adhesiolysis with the assistance of hyaluronidase. In another recent retrospective study, PRF was applied adjacent to the DRG at two different voltages (45 or 100 V) with the assistance of a multifunctional catheter in a population with low back pain, predominantly comprising patients with PSPS-II [20]. Although there was a notable reduction in pain scores across both groups, the high-voltage group exhibited a more pronounced decline in opioid consumption [20]. Although the data from our current study support the beneficial effects of EPRF application, as is the case with the existing literature, further studies are required in order to obtain high-quality evidence.

As previously stated, there are numerous theoretical advantages associated with the use of EPRF targeting the DRG. Firstly, in the context of EPRF application, the utilization of a multifunctional electrocatheter facilitates the delivery of stimulation in closer proximity to the DRG and provides the administration of medication to the epidural region [11]. Secondly, PRF can be applied to more than one DRG level via a single puncture on the skin, thereby enhancing the procedural comfort of patients scheduled for treatment at multiple levels. Another potential benefit is the ability to intervene in epidural fibrosis, which is frequently encountered in patients with PSPS-II, achieved through mechanical intervention with the assistance of a multifunctional catheter or the additional use of hyaluronidase. Furthermore, the relatively large area of the epidural space where the PRF is applied allows for the administration of higher volumes of medication with a reduced incidence of nerve irritation in comparison to the classical transforaminal approach. Further studies are required to examine these theoretically based possible advantages with advanced clinical studies, particularly to compare multifunctional catheter–mediated EPRF treatment with PRF adjacent to the DRG applied with the classical transforaminal approach. In addition to the possible advantages, the prolongation of the procedure time can be related to decreased patient comfort, especially in cases requiring more than 2 levels of application. The procedure can be performed in a shorter time with the classical transforaminal approach by using more than one needle. Although prolonged procedure time is not an absolute disadvantage, it is recommended that these two approaches be evaluated more deeply in terms of procedure duration and its potential effect on patient comfort.

The current study is subject to a number of limitations. The present study evaluates 3-month data as preliminary results, and 1-year follow-up of the patients continues. The patients were evaluated exclusively in terms of pain, and no multifaceted assessments were conducted, such as those pertaining to quality of life, disability, and mood. Further evaluations such as minimal clinically important difference to determine the clinical significance of the change in NRS score have not been performed. In accordance with ethical considerations, a comparison with the control group could not be undertaken. As hyaluronidase was not eligible for reimbursement by the healthcare system, it could not be administered to patients, thereby precluding the possibility of benefiting from its potential positive effects. Given the lack of clarity surrounding the optimal PRF treatment parameters, adjustments were made to ensure compatibility with existing literature. A salient issue that has been subject to deliberation pertains to the application of steroids and local anesthetics in conjunction with EPRF, with the objective of enhancing the clinical efficacy in the early phase. This approach serves to restrict the interpretation of the pure EPRF effect and the potential adverse impact of steroids on the PRF effect. Further well-designed studies comparing the possible effects of EPRF alone and the concomitant use of EPRF and steroids are required to clarify this debate. Besides all these limitations, our current study is valuable in terms of prospectively examining the effect of EPRF application on multifaceted pain assessment.

## 5. Conclusion

According to the preliminary results of the study, the application of EPRF adjacent to the DRG in patients with PSPS-II has been demonstrated to have a beneficial effect on the alleviation of pain, including neuropathic pain. Further investigation is required to elucidate the mechanism of action and clinical effectiveness of this approach. Furthermore, studies comparing the effects of EPRF with those of PRF adjacent to the DRG applied with the classical transforaminal approach are required.

## Figures and Tables

**Table 1:** Demographical and initial clinical information of the patients.

Gender (n)	F: 22 M: 20
Age (years) (SD)	50.2 (10.8)
Height (cm) (SD)	168.1 (9.6)
Weight (kg) (SD)	79.2 (18.8)
Initial symptom duration (months)	21.0 (7.8)
NRS (mean) (SD)	8.6 (1.4)
Presence of NP (n)	
With NP	23 (55%)
Without NP	19 (45%)
Drugs (n)	
Paracetamol	8
Paracetamol + codeine	18
NSAIDs	12
Gabapentinoids	36
Duloxetine	10
Tramadol	24

**Table 2:** Information about lumbar disc herniation operation (*operation characteristics*) and epidural pulsed RF intervention (*intervention characteristics*).

Operation characteristics	
Post-operation duration (months) (SD)	12.0 (4.8)
Presence of stabilization (n)	
Yes	8 (19%)
No	34 (81%)
Operation level (n)	
L1-2	1
L2-3	2
L3-4	12
L4-5	34
L5-S1	39

**Intervention characteristics**	

Dominant symptomatic side (n)	R: 20 L:22
Total number of target level(s) (n)	
L3-4	12
L4-5	30
L5-S1	40
Target level(s) in one patient (n)	
One level	9
Two levels	26
Three levels	7

**Table 3:** Time-dependent change in NRS scores and number of the patients with neuropathic pain according to DN-4 scale.

NRS	Mean (SD)		*p* value
Baseline	8.6 (1.4)		
Month 1	3.0 (0.4)		< 0.001
Month 3	4.0 (0.4)		< 0.001

**DN-4**	**NP (+)**	**NP (−)**	**p** **value**
**Baseline**	**23**	**19**	

Month 1	10	32	< 0.001
Month 3	22	20	0.317

*Note:* “+” sign indicates the presence of NP.

Abbreviation: NP, neuropathic pain.

## Data Availability

The datasets used/analyzed in the current study are available from the corresponding author upon reasonable request.
